# Magnetoencephalographic source imaging improves localization of the epileptogenic zone in multimodal imaging evaluation

**DOI:** 10.1002/epi.70359

**Published:** 2026-06-26

**Authors:** Romain Bouet, Marc Hermier, Karine Ostrowsky‐Coste, Sebastien Daligault, Sandrine Bouvard, Hélène Catenoix, Alexandra Montavont, Sébastien Boulogne, François Mauguière, Sylvain Rheims, Julien Jung

**Affiliations:** ^1^ Lyon Neuroscience Research Center, Inserm U1028, CNRS UMR5292 Lyon 1 University Lyon France; ^2^ Department of Neuroradiology Hospices Civils de Lyon Lyon France; ^3^ Department of Paediatric Epileptology Woman‐Mother‐Child Hospital Hospices Civils de Lyon Lyon France; ^4^ MEG Department CERMEP‐Imagerie du Vivant Lyon France; ^5^ Department of Functional Neurology and Epileptology Hospices Civils de Lyon and Lyon 1 University Lyon France; ^6^ Idée Epilepsy Institute Lyon France

**Keywords:** epilepsy surgery, magnetoencephalography, MRI, PET, SEEG

## Abstract

**Objective:**

To assess the added diagnostic value of magnetoencephalographic source imaging (MSI) beyond conventional magnetic resonance imaging (MRI) and fluorodeoxyglucose–positron emission tomography (FDG‐PET) in localizing the epileptogenic zone, with stereo‐electroencephalography (SEEG) and surgical resection serving as reference standards.

**Methods:**

Among 101 patients prospectively enrolled with drug‐resistant focal epilepsy in the EPIMAGE study to assess the diagnostic yield of non‐invasive multimodal imaging (MSI, MRI, FDG‐PET), those undergoing SEEG and/or surgery were analyzed to compare imaging with SEEG seizure‐onset zone (SOZ) or resection zone.

We employed binomial generalized linear mixed models (GLMMs) to evaluate the detection accuracy of MRI, PET, and MSI, individually and in combination, for identifying the SEEG‐defined SOZ and resection zones. We further assessed the spatial concordance between imaging abnormalities and these reference standards.

**Results:**

Using SEEG as reference, MSI identified additional SOZ regions beyond MRI in 55.6% of patients, beyond PET in 29.6%, and beyond combined MRI + PET in 33.3%. When the resection zone served as reference, MSI added new localizing value beyond MRI in 55.6% of patients, beyond PET in 29.6%, and beyond MRI + PET in 33.3%.

GLMM analysis revealed that bimodal combinations improved detection rates (MRI + PET: 73.7%; MRI + MSI: 81.3%; PET+MSI: 79.8%). The trimodal combination (MRI + PET+MSI) achieved the highest detection probability at 85.3%, significantly higher than MRI, PET, or MSI alone (*p* = .003, *p* = .008, and *p* = .008, respectively). MSI significantly enhanced SOZ detection when added to either MRI (*p* = .01) or PET (*p* = .05).

**Significance:**

MSI adds significant localizing value to the presurgical workup of focal epilepsy by expanding the detection of the epileptogenic network beyond the limits of structural MRI and metabolic PET. The primary clinical benefit of MSI lies in its ability to identify additional seizure‐onset regions in complex cases, without increasing false‐positive localizations. These findings demonstrate that a multimodal approach incorporating MSI optimizes surgical planning by increasing the probability of capturing the full extent of the epileptogenic zone.

**Key points**

- At the patient level, magnetoencephalographic source imaging (MSI), positron emission tomography (PET), and magnetic resonance imaging (MRI) show similar concordance with the stereo‐electroencephalography (SEEG) seizure‐onset zone and the resection zone.
- Adding MSI to MRI and/or PET significantly increases seizure onset– and resection‐zone detection.
- Multimodal union improves sensitivity, whereas modality overlap yields high specificity.
- MSI adds complementary information for localizing the epileptogenic cortex, especially in complex or MRI‐negative cases.

## 1 INTRODUCTION

Presurgical evaluation of drug‐resistant focal epilepsy requires the integration of clinical, electrophysiological, and imaging data to accurately define the epileptogenic zone, the cortical area whose resection is necessary to achieve seizure freedom.^1^, ^2^ High‐resolution structural magnetic resonance imaging (MRI) and interictal fluorodeoxyglucose–positron emission tomography (FDG‐PET) are used routinely to identify lesions or hypometabolic regions that can guide intracranial exploration and surgical planning.^2^, ^3^

Magnetoencephalographic source imaging (MSI), which applies source localization algorithms to magnetoencephalography (MEG) recordings, has emerged as a valuable non‐invasive tool for localizing interictal epileptiform discharges with high spatiotemporal resolution.^4^, ^5^, ^6^ MSI plays an increasingly strategic role in stereo–electroencephalography (SEEG) planning by refining implantation hypotheses^5^, ^7^, ^8^, ^9^, ^10^ to influence candidate selection for SEEG. A focal, plausible MSI source may support the decision to proceed with invasive monitoring, whereas non‐localizing MSI findings might prompt alternative management.^9^

Despite its growing clinical use, MSI is still often viewed as a second‐line investigation. Its precise added value compared to MRI and PET remains unclear, and it is not systematically integrated into presurgical workflows. Whether MSI provides truly complementary information or simply confirms findings from other modalities has not been rigorously assessed.^11^

This study aims to determine the specific contribution of MSI by comparing its localizing value to that of MRI and PET in a cohort of patients who underwent SEEG. We evaluate whether MSI identifies regions overlapping with SEEG‐defined seizure‐onset zones (SOZs) and resected tissue, and whether it offers additive information. Notably, MRI and PET were analyzed using both standard visual inspection and advanced post‐processing: morphometric analysis programs (MAP) for MRI,^12^, ^13^ and statistical parametric mapping (SPM) for PET.^14^ We hypothesize that MSI provides added value for localizing the SOZ and resected area, as it captures a specific electrophysiological signature of epilepsy complementary to structural MRI lesions or metabolic PET abnormalities.

Standard protocol approvals, registrations, and patient consents.

Ethics Committee agreement was obtained for the study (CCP Lyon Sud Est IV Research Ethics Committee, approval number: N2012‐A00516‐37, 05/24/2012) in accordance with the Declaration of Helsinki. Patients were fully informed and signed a written consent form.

## 2 MATERIALS AND METHODS

### 2.1 Study design and population

The patients included in this study were enrolled in the prospective, monocentric observational EPIMAGE study (Multimodal Imaging in the Presurgical Evaluation of Drug‐Resistant Focal Epilepsy, NCT01735032).

The primary objective of EPIMAGE was to assess the value of non‐invasive multimodal imaging (MRI, PET, and MSI) for localizing the epileptogenic zone in patients considered for epilepsy surgery. Inclusion criteria were: (i) adults with drug‐resistant focal epilepsy for more than 2 years; (ii) candidacy for epilepsy surgery; and (iii) presence of interictal epileptiform discharges documented during scalp EEG monitoring, irrespective of their frequency or localization.

**FIGURE 1:**
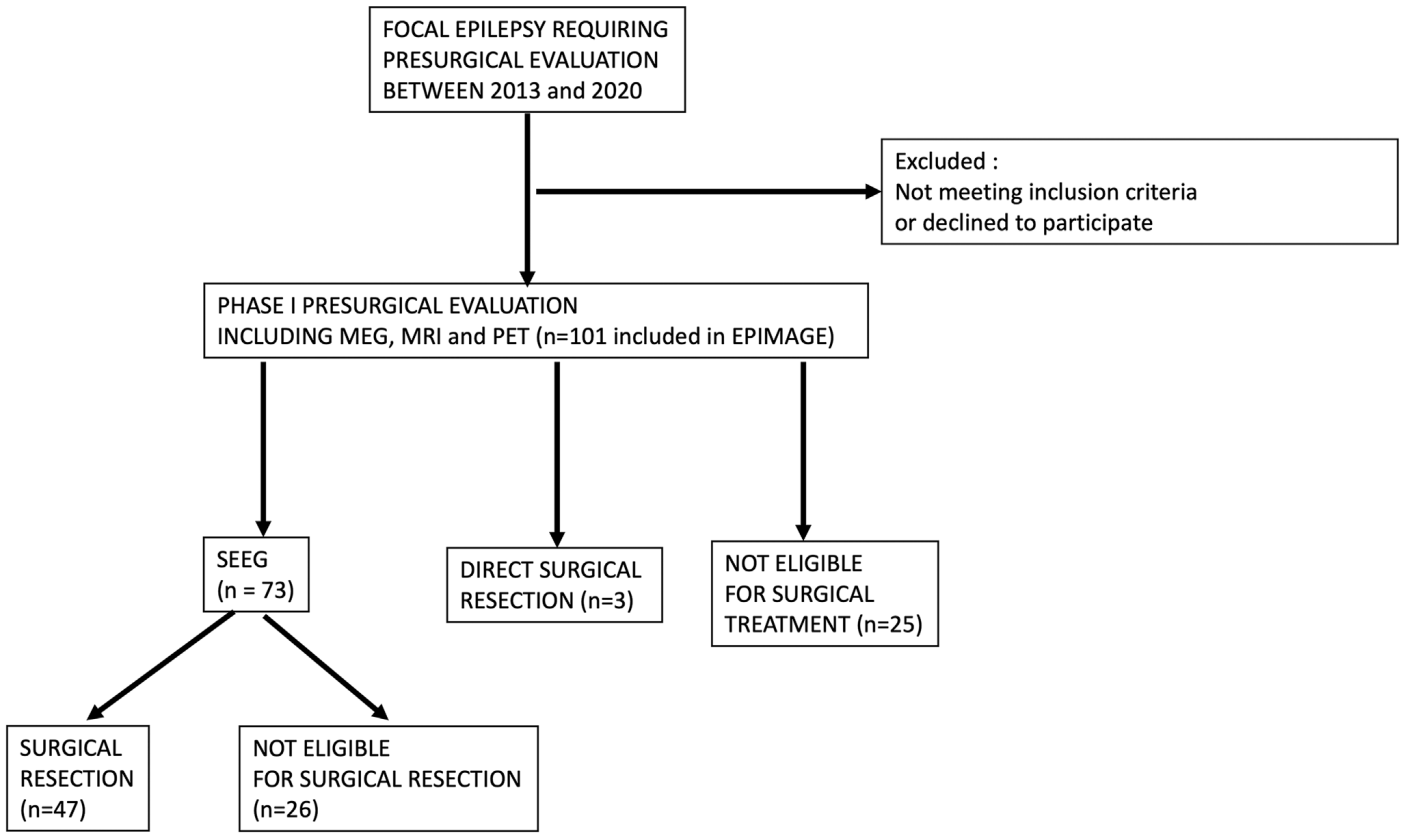
Flowchart of the EPIMAGE study.

From the EPIMAGE cohort, we selected patients who underwent SEEG or direct surgical resection to enable comparison of noninvasive imaging findings with the SEEG‐defined SOZ or the resected cortex. A flowchart of the study is presented in Figure 1.

### 2.2 Multimodal neuro‐imaging data acquisition

Each patient included in the EPIMAGE study underwent a standard non‐invasive presurgical work‐up including prolonged scalp EEG monitoring, brain MRI,^15^ FDG‐PET scan, and interictal MSI. MRI and PET were visually analyzed by neuroradiologists or epileptologists trained in the identification of epileptogenic abnormalities (M.H. for MRI, K.O.C. for FDG PET). MSI data were analyzed by two experienced readers (R.B. and J.J.). Statistical post‐processing techniques of MRI and PET data were also applied to assist in surgical planning.

The complete multimodal non‐invasive dataset was then integrated during a multidisciplinary epilepsy surgery conference to formulate a working hypothesis of the epileptogenic zone, to guide SEEG implantation and surgical planning, or to conclude to a contraindication to surgery in cases of marked discordance between non‐invasive findings.

Details for acquisition of multimodal data are provided in Supplementary Material (Methods S1).

### 2.3 Region of interest (ROI)–based standardization across neuroimaging modalities

Brain MRI, PET, and source modeling in MSI have different spatial resolutions. The statistical post‐processing applied to these images results in maps with clusters of different sizes. In contrast, the limited spatial sampling of SEEG electrodes does not allow for the construction of volumetric maps of the SOZ that are directly comparable to neuroimaging results.

To enable a meaningful comparison of all neuroimaging maps and SEEG with a reasonable anatomic level of detail, we mapped all significant clusters from MRI, PET, and MSI onto 18 predefined anatomic subregions for each hemisphere. The regions of interest (ROIs) used in this study are detailed in the Supplementary Material (Methods S2).

ROIs for MRI and PET correspond to regions identified through both expert visual assessment and statistical post‐processing; MSI ROIs were defined using the volumetric imaging of epileptic spikes (VIES) method,^10^ and SEEG SOZ was determined by expert visual analysis of ictal recordings. Details for analysis of MRI, PET, MSI, and SEEG data analysis are provided in Supplementary Material (Methods S2).

### 2.4 Localizing value of multimodal neuroimaging data for SEEG SOZ determination and resection zone

#### 2.4.1 Added value of MSI for identifying the SOZ and the resected zone at the patient level

To assess the added value of MSI in presurgical evaluation, we compared MSI results with those of other imaging modalities (MRI, PET, and their combination) in identifying (1) the SOZ and (2) the resected brain area. For each comparison and each patient, we determined the proportion of patients for whom MSI provided added, redundant, or discordant information relative to another modality, based on the following definitions: (i) ADD: MSI identified at least one additional region not detected by the other modality, and this region was part of the SOZ or resected area. (ii) REDUNDANT: MSI identified only regions already detected by the other modality, and all were located within the SOZ or resected area. (iii) DISCORDANT: MSI identified regions that were not part of the SOZ or resected area.

#### 2.4.2 ROI‐level diagnostic performance

Diagnostic performance was assessed at the ROI level using SEEG‐defined SOZ or the surgical resection zone as reference. ROIs were classified as true positives when overlapping with the reference zone, false positives when located outside it, false negatives when belonging to the reference zone but undetected, and true negatives when outside the reference zone and not identified as pathological.

In addition to individual modalities (MRI, PET, and MSI), we evaluated combinations of imaging results. For each pair or group of modalities, we generated a union volume, defined as all brain regions identified by at least one of the modalities. We also evaluated intersection volumes, defined as the set of regions identified by all modalities in the combination.

Standard diagnostic metrics (sensitivity, specificity, positive predictive value [PPV], and negative predictive value [NPV]) were calculated at the ROI level for each modality and combination of modalities. To ensure statistical robustness and address the limitations of small sample sizes in specific subgroups, these metrics were computed by pooling data across all informative patients (micro‐average approach). We estimated 95% confidence intervals (95% CIs) using the Clopper‐Pearson exact binomial method. Of note, these metrics were calculated exclusively for informative cases; for each imaging modality and combination, strictly negative scans were excluded from this specific analysis.

Sensitivity defined as the proportion of true SOZ regions correctly identified by a given modality, reflects the ability to detect all epileptogenic regions while minimizing the risk of missing true SOZ areas. The PPV, defined as the proportion of regions identified as positive that truly belong to the SOZ, reflects the spatial precision of a modality, indicating how reliably a positive finding corresponds to a true epileptogenic region. The F1‐score, defined as the harmonic mean of sensitivity and PPV, was also computed as a composite measure of diagnostic performance. By jointly accounting for both the completeness of SOZ detection and the spatial precision of the identified regions, the F1‐score provides a balanced summary metric particularly suited to evaluating multimodal combinations, where gains in sensitivity may otherwise be offset by reductions in PPV.

#### 2.4.3 Statistical analysis of SOZ detection by neuroimaging modalities and congruence with SEEG

All statistical analyses were performed using R version 3.6.1. Generalized linear mixed models (GLMMs) were fitted using the lme4 package and estimated marginal means were computed using the emmeans package.

We used GLMMs with a binomial distribution to compare the ability of each imaging modality (MRI, PET, MSI), and their combinations, to detect the SOZ as defined by SEEG.

Each SEEG ROI was classified as either “detected” (if it overlapped with at least one region identified by the imaging modality) or “not detected.” These binary outcomes were modeled using GLMMs, with modality as a fixed effect and subject as a random effect. MRI was used as the reference modality. Detection probabilities, defined as the GLMM‐estimated likelihood that a given modality correctly identifies a SOZ region, were estimated exclusively in patients with at least one positive imaging finding for the modality of interest, and their 95% CIs were reported. Post hoc pairwise comparisons between modalities were performed using estimated marginal means, with false discovery rate (FDR) correction.

Then, a second analysis was performed to assess, for each modality or combination of modalities, the proportion of identified regions that were congruent with the SEEG‐defined SOZ. This analysis was similarly restricted to informative cases, defined as patients in whom the modality identified at least one positive region. An imaging ROI was defined as congruent when it spatially overlapped with at least one SEEG ROI belonging to the SOZ. Congruence probability, defined as the GLMM‐estimated likelihood that a region identified by a given modality truly belongs to the SOZ, thus reflects the spatial precision of each modality rather than its detection capacity. For combined modalities, we also evaluated intersection volumes, defined as the set of regions identified by all modalities in the combination.

It should be noted that post hoc pairwise comparisons between combinations span modalities with different effective sample sizes, given that MSI analyses were necessarily restricted to the subset of patients with recordable interictal spikes.

Both analyses (detection and congruence) were conducted first across all patients, and then repeated in the subgroup of patients who underwent surgery and achieved seizure freedom with at least 1 year follow‐up (Engel class I).

#### 2.4.4 Statistical analysis of neuroimaging performance in detecting and localizing the resection zone

The same detection and congruence analyses were performed using the resected volume instead of the SEEG‐defined SOZ, to evaluate the relationship between imaging findings and the resected regions. These analyses were conducted across all patients, and then repeated in the subgroup of patients who achieved seizure freedom (Engel class I).

## 3 RESULTS

### 3.1 Characteristics of patients and epilepsy and diagnostic yield of neuroimaging investigations

Between January 2013 and December 2020, a total of 101 patients were enrolled in the EPIMAGE study at Lyon University Hospital. Among them, 73 underwent SEEG, of whom 47 subsequently had surgery (SEEG_surgery group) and 26 did not (SEEG_no_surgery group). In addition, three patients underwent direct surgery without prior SEEG (Table 1).

**TABLE 1:** Descriptive statistics of patient subgroups.

	SEEG followed by cortectomy (*N* = 47)	SEEG without cortectomy (*N* = 26)	Direct surgery without SEEG (*N* = 3)
Epilepsy duration, years, mean ± SD	18.4 ± 9.88	23.08 ± 13.17	16.23 ± 11.82
Age at MSI recording, years, mean ± SD	32.86 ± 8.24	33.14 ± 11.82	46.98 ± 1.55
Positive MSI findings [95% CI]	0.55 [0.4–0.7]	0.54 [0.34–0.73]	0.33 [0.02–0.87]
Positive MRI findings [95% CI]	0.74 [0.59–0.86]	0.77 [0.56–0.90]	1
Positive PET findings [95% CI]	0.81 [0.66–0.9]	0.73 [0.52–0.90]	1
MSI localized ROI, mean ± SD	2.19 ± 0.83	2.07 ± 0.92	2
MRI localized ROI, mean ± SD	1.69 ± 0.9	2 ± 1.08	1.33 ± 0.58
PET localized ROI, mean ± SD	1.84 ± 1	2.16 ± 0.90	1.33 ± 0.58
SEEG localized ROI, mean ± SD	2.43 ± 0.83	2.42 ± 1.06	NA
Resected regions, mean ± SD	2.26 ± 0.74	NA	1.67 ± 0.58
Proportion of Engel class I outcome [95% CI]	0.59 [0.43–0.73]	NA	1
Epilepsy type proportion [95% CI]	mTLE 0.23 [0.13–0.38] TLE 0.3 [0.18–0.45] EXTLE 0.47 [0.32–0.68]	mTLE 0 TLE 0.35 [0.18–0.56] EXTLE 0.65 [0.44–0.82]	mTLE 0.33 [0.02–0.87] TLE 0.47 [0.18–0.56] EXTLE 0.67 [0.13–0.98]

*Note*: Summary of demographic, clinical, imaging, and outcome data for three patient groups: (1) patients who underwent SEEG followed by surgical resection, (2) patients who underwent SEEG without following surgery, and (3) patients who were directly operated without preceding SEEG. Continuous variables are expressed as mean ± standard deviation (SD). Imaging positivity rates (MRI, PET, MSI), Engel class I outcome rates, and proportions of epilepsy type (mTLE: medial temporal lobe epilepsy/TLE: lateral temporal lobe epilepsy/EXTLE: extra‐temporal lobe epilepsy) are reported with their 95% confidence intervals. Zone counts refer to the number of distinct anatomic regions identified by each modality (or intervention).

The mean age at the time of MSI recording was 33 years in both SEEG_surgery group and SEEG_no_surgery group, and 47 years in the direct surgery group. Medial temporal lobe epilepsy represented only a minority of cases across groups: 23% in SEEG_surgery, absent in SEEG_no_surgery, and 33% in direct surgery. Most patients presented with lateral temporal lobe epilepsy (30% in SEEG_surgery, 35% in SEEG_no_surgery), or, more frequently, extra temporal lobe epilepsy (47% in SEEG_surgery, 65% in SEEG_no_surgery, 67% in direct surgery).

MSI was positive in 55% of patients in both SEEG groups, compared with 33% of those undergoing direct surgery. MRI was positive in 75% of SEEG patients (59% on visual analysis and an additional 16% only after statistical post‐processing) and in 100% of direct surgery patients. PET was positive in 73% of SEEG_no_surgery patients and 81% of SEEG_surgery patients, with no additional yield from statistical post‐processing. PET was also positive for all patients in the direct surgery group. The number of ROIs identified was consistent across modalities: MSI localized ~2 ROIs in both SEEG groups; MRI and PET localized ~1.7–2 ROIs on average; and SEEG identified ~2.4 ROIs within the seizure‐onset zone. Regarding the spatial overlap between MSI ROI and SEEG sampling, in the subgroup of 48 MSI‐positive patients who underwent SEEG, 88% (±0.29 standard deviation [SD]) of the MSI ROIs were effectively explored by SEEG electrodes. However, these specific targets accounted for only 16% (±0.12 SD) of the total ROIs implanted per patient.

In the SEEG_surgery group, 59% of patients achieved an Engel class I outcome, whereas all three patients in the direct surgery group were seizure‐free.

Comprehensive patient and imaging details are provided in Table S1.

### 3.2 Added value of MSI for identifying the SOZ and the resected zone at the patient level

Using SEEG as reference (Table 2), MSI identified additional SOZ regions in 55.6% of patients vs MRI, 29.6% vs PET, and 33.3% vs MRI ∪ PET, while providing redundant information in 37%, 62.9%, and 59.3% of patients, respectively. These proportions were similar in patients with Engel class 1 outcome.

**TABLE 2:** Classification of the added value of MSI in identifying either the seizure‐onset zone (SOZ) or the resected area, compared to MRI, PET, and the union of both (MRI ∪ PET).

Comparison	Add (%)	Redundant (%)	Discordant (%)
SOZ (All): MSI vs MRI	55	35	10
SOZ (All): MSI vs PET	42.5	47.5	10
SOZ:(All): MSI vs MRI ∪ PET	37.5	52.5	10
SOZ (Engel 1): MSI vs MRI	60	33	6.7
SOZ (Engel 1): MSI vs PET	26.7	66.7	6.7
SOZ:(Engel 1): MSI vs MRI ∪ PET	26.7	66.7	6.7
Resection zone (All): MSI vs MRI	55.6	37	7.4
Resection zone (All): MSI vs PET	33.3	59.3	7.4
Resection zone (All): MSI vs MRI ∪ PET	29.6	63	7.4
Resection zone (Engel 1): MSI vs MRI	56.2	37.5	6.2
Resection zone (Engel 1): MSI vs PET	25.0	68.8	6.2
Resection zone (Engel 1): MSI vs MRI ∪ PET	25.0	68.8	6.2

*Note*: For each comparison, patients were categorized as: ADD: MSI identified additional regions not visible with the reference modality, and these regions overlapped with the SOZ or resected zone — suggesting a true added value /REDUNDANT: MSI localizations overlapped with those of the reference modality, without adding new informative regions / DISCORDANT: MSI localizations did not overlap with the SOZ or resected area. Added value was computed on informative cases only, defined as patients MSI positive. Values are expressed as percentages of the patients with analyzable data for each comparison. Results are presented for the whole group of patients (All) and for the subgroup of patients seizure‐free (Engel class I).

Using resection zone as reference (Table 3), compared to MRI, MSI identified additional regions in 55.6% of patients, showed redundant results in 37%, and discordant findings in 7.4%. When compared to PET, MSI added new localizing value in 29.6%, provided redundant information in 62.9%, and was discordant in 7.4%. Finally, relative to the combined MRI and PET data, MSI pinpointed additional SOZ regions in 33.3%, was redundant in 59.3%, and discordant in 7.4%. The same repartition was seen in the subgroup of patients Engel class 1.

**TABLE 3:** Diagnostic performance of single and combined imaging modalities relative to SEEG and surgical outcome.

Modality	Reference	Sensitivity	Specificity	PPV	NPV	F1‐score
MSI	SEEG SOZ	0.615 [0.515–0.709]	0.984 [0.975–0.990]	0.744 [0.639–0.832]	0.970 [0.960–0.979]	0.674 [0.602–0.740]
MSI	Resection zone	0.677 [0.547–0.791]	0.981 [0.970–0.989]	0.712 [0.579–0.822]	0.978 [0.966–0.987]	0.694 [0.604–0.775]
PET	SEEG SOZ	0.607 [0.520–0.690]	0.985 [0.978–0.990]	0.739 [0.647–0.818]	0.973 [0.964–0.979]	0.667 [0.604–0.725]
PET	Resection zone	0.705 [0.598–0.797]	0.991 [0.985–0.996]	0.838 [0.734–0.913]	0.981 [0.973–0.988]	0.765 [0.693–0.828]
MRI	SEEG SOZ	0.591 [0.502–0.676]	0.989 [0.983–0.993]	0.788 [0.694–0.864]	0.971 [0.963–0.978]	0.675 [0.611–0.735]
MRI	Resection zone	0.675 [0.563–0.774]	0.995 [0.989–0.998]	0.889 [0.784–0.954]	0.979 [0.970–0.986]	0.767 [0.690–0.833]
MRI ∪ PET	SEEG SOZ	0.642 [0.562–0.716]	0.985 [0.979–0.989]	0.750 [0.669–0.820]	0.975 [0.967–0.981]	0.692 [0.635–0.744]
MRI ∪ PET	Resection zone	0.728 [0.632–0.811]	0.991 [0.985–0.995]	0.843 [0.750–0.911]	0.983 [0.975–0.988]	0.781 [0.716–0.838]
MRI ∪ MSI	SEEG SOZ	0.699 [0.620–0.769]	0.981 [0.974–0.986]	0.727 [0.648–0.796]	0.978 [0.971–0.984]	0.712 [0.658–0.762]
MRI ∪ MSI	Resection zone	0.789 [0.694–0.866]	0.984 [0.976–0.990]	0.765 [0.669–0.845]	0.986 [0.979–0.992]	0.777 [0.712–0.834]
PET ∪ MSI	SEEG SOZ	0.686 [0.607–0.758]	0.977 [0.970–0.983]	0.686 [0.607–0.758]	0.977 [0.970–0.983]	0.686 [0.631–0.737]
PET ∪ MSI	Resection zone	0.771 [0.674–0.850]	0.981 [0.972–0.987]	0.718 [0.621–0.803]	0.985 [0.978–0.991]	0.744 [0.677–0.803]
MRI ∪ PET ∪ MSI	SEEG SOZ	0.735 [0.661–0.800]	0.977 [0.970–0.982]	0.693 [0.619–0.760]	0.981 [0.974–0.986]	0.713 [0.662–0.761]
MRI ∪ PET ∪ MSI	Resection zone	0.825 [0.738–0.893]	0.981 [0.973–0.987]	0.739 [0.649–0.817]	0.989 [0.982–0.993]	0.780 [0.719–0.833]
MRI ∩ PET	SEEG SOZ	0.552 [0.452–0.650]	0.989 [0.983–0.994]	0.784 [0.673–0.871]	0.970 [0.960–0.978]	0.648 [0.573–0.718]
MRI ∩ PET	Resection zone	0.632 [0.507–0.746]	0.995 [0.989–0.999]	0.896 [0.773–0.965]	0.977 [0.967–0.985]	0.741 [0.652–0.818]
MRI ∩ MSI	SEEG SOZ	0.559 [0.424–0.688]	0.998 [0.991–1.000]	0.943 [0.808–0.993]	0.969 [0.954–0.979]	0.702 [0.599–0.792]
MRI ∩ MSI	Resection zone	0.590 [0.421–0.744]	0.998 [0.991–1.000]	0.958 [0.789–0.999]	0.974 [0.959–0.985]	0.730 [0.603–0.834]
PET ∩ MSI	SEEG SOZ	0.609 [0.479–0.729]	0.998 [0.992–1.000]	0.951 [0.835–0.994]	0.973 [0.961–0.983]	0.743 [0.648–0.823]
PET ∩ MSI	Resection zone	0.714 [0.554–0.843]	1.000 [0.995–1.000]	1.000 [0.884–1.000]	0.983 [0.970–0.991]	0.833 [0.727–0.911]
MRI ∩ PET ∩ MSI	SEEG SOZ	0.571 [0.422–0.712]	0.997 [0.990–1.000]	0.933 [0.779–0.992]	0.971 [0.956–0.982]	0.709 [0.596–0.806]
MRI ∩ PET ∩ MSI	Resection zone	0.600 [0.421–0.761]	1.000 [0.993–1.000]	1.000 [0.839–1.000]	0.975 [0.958–0.986]	0.750 [0.616–0.856]

*Note*: Values are presented as proportions with 95% confidence intervals [95% CIs]. Performance metrics were computed exclusively on informative cases, defined as patients MSI positive. Gold Standards: Comparisons were made against the seizure‐onset zone defined by stereoelectroencephalography (vs SEEG) and the resection zone (vs resection zone). Combined Strategies: “Union” (e.g., MRI ∪ MSI) refers to the identification of the zone by at least one of the modalities. “Intersection” (e.g., MRI ∩ MSI) refers to spatial concordance between the modalities.

Abbreviations: MRI, magnetic resonance imaging; MSI, magnetoencephalography source imaging; NPV, negative predictive value; PET, positron emission tomography; PPV, positive predictive value.

### 3.3 ROI‐level diagnostic performance of multimodal neuroimaging

Diagnostic performances are presented in Table 3.

Relative to SEEG, MSI demonstrated a sensitivity of 61.5% and a PPV of 74.4% against the SEEG standard, comparable to MRI and PET. Higher performance was observed against the surgical standard, with sensitivities ranging between 67.5% and 70.5%, and PPVs varying from 71.2% to 88.9%.

Using a union approach, adding MSI increased sensitivity compared to single modalities Relative to SEEG, the MRI + MSI combination achieved a sensitivity of 70.0% (compared to 59.1% for MRI alone), while maintaining a comparable PPV (72.7% vs 78.8%). Similarly, the PET + MSI union (68.6%) surpassed PET alone (60.7%). This gain in sensitivity without a major loss of precision was confirmed against the surgical standard, with the MRI + MSI union reaching 78.9% sensitivity vs 67.5% for isolated MRI. Maximal sensitivity was obtained by the triple union including MSI (MRI + PET + MSI), reaching 73.5% vs SEEG and 82.5% vs surgery. Of note, the F1‐score was either stable or increased in all combinations including MSI compared to MRI and PET alone, with the notable exception of the PET + MSI union compared to PET alone for the resection zone, where a sharp loss of spatial precision caused the F1‐score to decrease. Furthermore, the triple unions consistently yielded increased overall classification scores. This suggests that for most combinations, the substantial gain in sensitivity was achieved without a severe drop in PPV.

Using an intersection approach, the confirmation of a focus by MSI reinforced PPV compared to single modalities. Against the surgical standard, the MRI ∩ MSI concordance presented a PPV of 95.8% (vs 88.9% for MRI alone), and the PET ∩ MSI concordance achieved a perfect PPV of 100% (vs 83.8% for PET alone).

#### 3.3.1 Statistical analysis of SOZ detection by neuroimaging modalities and congruence with SEEG

**FIGURE 2:**
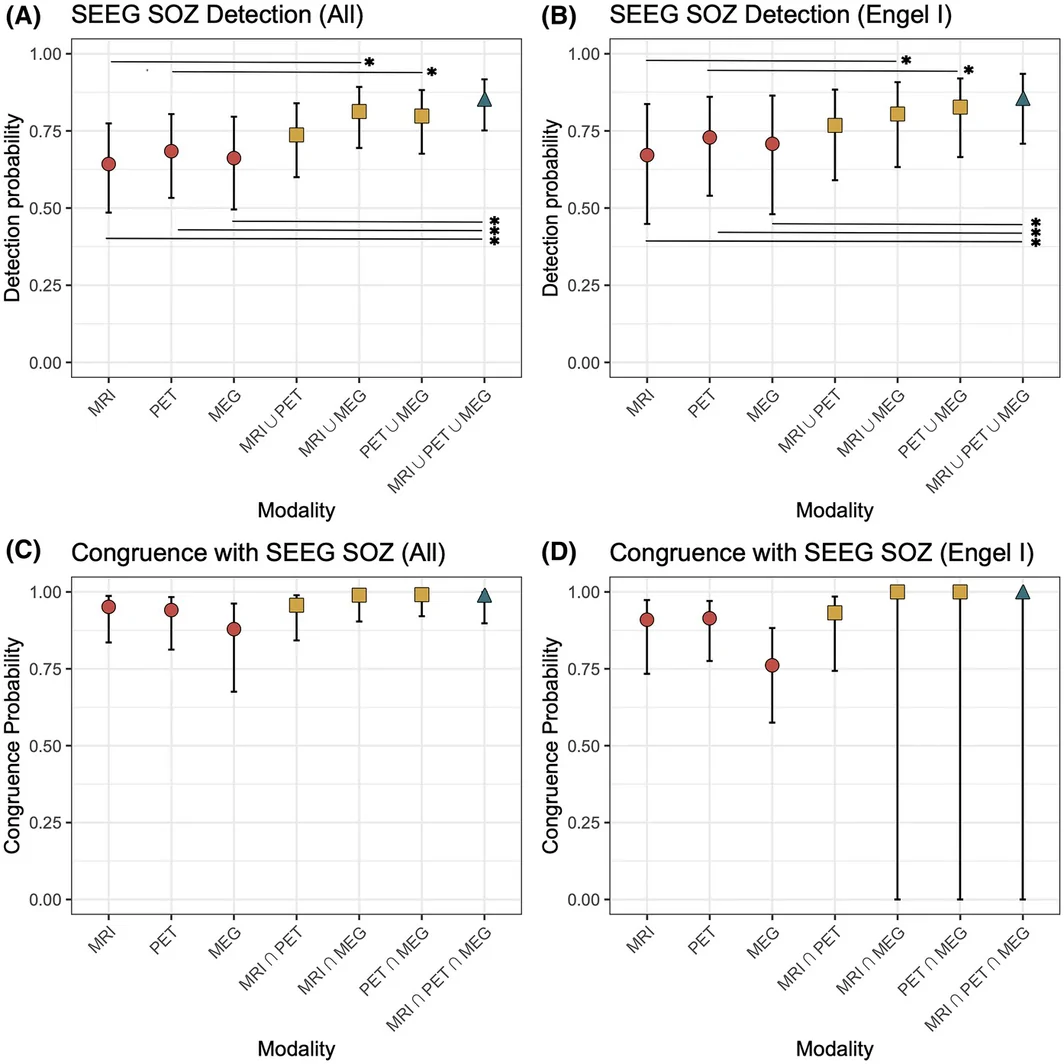
Detection and congruence of imaging modalities with the seizure‐onset zone (SOZ) at the ROI level. (A) Probability of detecting SEEG‐defined SOZ ROIs by individual or combined imaging modalities (MRI, PET, MSI, and unions) in the full patient cohort. (B) Same as (A), restricted to patients with good post‐surgical outcome (Engel class I). (C) Congruence between individual or combined imaging modalities (MRI, PET, MSI, and intersections) and SEEG‐defined SOZ ROIs in the full cohort. (D) Same as (C), restricted to patients with good outcome. Estimated marginal means from logistic mixed‐effects models are shown with 95% confidence intervals (CIs). In the plots, single modalities are represented by red circles, combinations of two modalities (union or intersection) by yellow squares, and the combination of all three modalities by green triangles. Detection was defined as an overlap between the imaging‐defined regions and SEEG‐defined SOZ ROIs. Congruence was coded when the SEEG‐defined SOZ ROI was included in the imaging‐defined region. All models included random intercepts for subjects. Horizontal lines with an asterisk indicate statistically significant post hoc comparisons between imaging modalities for detection and congruence. The wide CIs observed for some intersection‐based modalities in the Engel class I subgroup (D) reflect the reduced sample size in this subset, which limits the stability of model estimates and the precision of the marginal means, and should therefore be interpreted with caution.

Post hoc analyses of the estimated detection rates of the SOZ showed that the combination of MRI, PET, and MSI was significantly superior to MRI alone (*p* = .003), PET alone (*p* = .008), and MSI alone (*p* = .008). The addition of MSI to either MRI or PET (MRI ∪ MSI and PET ∪ MSI) also significantly improved detection rates compared with each modality alone (*p* = .01 and *p* = .05, respectively). Similar findings were obtained when restricting the analyses to patients with an Engel class I surgical outcome (Figure 2B). Detailed statistical results are provided in Table S2.

The estimated probabilities of congruence between the regions identified by imaging and the SEEG‐defined SOZ were high across all single and combined modalities (Figure 2C), above 90%. Although the triple combination MRI ∩ PET ∩ MSI yielded the highest congruence estimate (99%), no statistically significant differences were observed between modality combinations after correction for multiple comparisons, likely reflecting a ceiling effect given the consistently high congruence rates across all modalities. The same pattern was observed when the analysis was restricted to patients achieving an Engel class I outcome (Figure 2D). Detailed statistical results are provided in Table S3.

(See Figure S2 for an example of multimodal convergence in a representative patient).

#### 3.3.2 Statistical analysis of neuroimaging performance in detecting and localizing the resection zone

**FIGURE 3:**
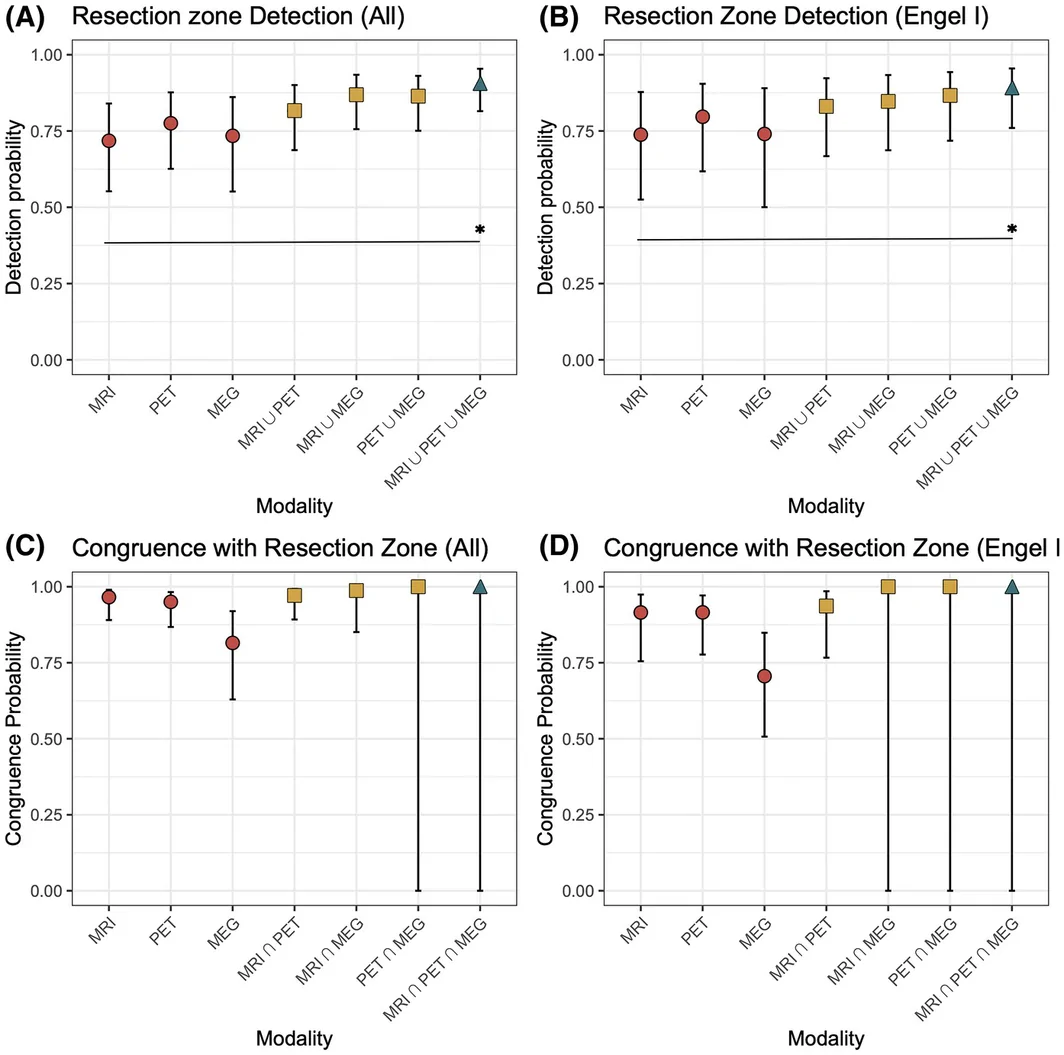
Detection and congruence of imaging modalities with the resection zone at the region of interest (ROI) level. (A) Probability of detecting resection zone‐defined ROIs by individual or combined imaging modalities (MRI, PET, MSI, and unions) in the full patient cohort. (B) Same as (A), restricted to patients with good post‐surgical outcome (Engel class I). (C) Congruence between individual or combined imaging modalities (MRI, PET, MSI, and intersections) and resection zone ROIs in the full cohort. (D) Same as (C), restricted to patients with good outcome. Estimated marginal means from logistic mixed‐effects models are shown with 95% confidence intervals (CIs). In the plots, single modalities are represented by red circles, combinations of two modalities (union or intersection) by yellow squares, and the combination of all three modalities by green triangles. Detection was defined as an overlap between the imaging‐defined regions and resection zone ROIs. Congruence was coded when the resection zone ROI was included in the imaging‐defined region. All models included random intercepts for subjects. Horizontal lines with an asterisk indicate statistically significant post hoc comparisons between imaging modalities for detection and congruence. The wide CIs observed for some intersection‐based modalities in the Engel class I subgroup (B and D) reflect the reduced sample size in this subset, which limits the stability of model estimates and the precision of the marginal means, and should therefore be interpreted with caution.

Detection probabilities for the resection zone were comparable across individual modalities (between 72% and 77%). Combining modalities progressively increased detection probability, reaching 90% with the full MRI ∪ PET ∪ MSI combination. However, post hoc testing revealed only one statistically significant difference after correction for multiple comparisons: MRI alone vs MRI ∪ PET ∪ MSI (*p* = .02). Similar findings were obtained when restricting the analysis to patients with an Engel class I outcome (Figure 3B), and detailed statistical results are provided in Table S4.

Congruence probabilities between imaging modalities and the surgical resection zone were uniformly high (above 80%) across all single and combined modalities (Figure 3C), with the triple combination MRI ∩ PET ∩ MSI reaching a perfect congruence of 100%. No statistically significant differences were observed after correction for multiple comparisons, likely reflecting a ceiling effect given the consistently high congruence rates. The same pattern was observed when the analysis was restricted to patients with an Engel class I outcome (Figure 3D). Detailed statistical results are provided in Table S5.

### 3.4 Localization accuracy of unimodal imaging modalities for SEEG SOZ and resection zone determination at the patient level

Finally, we examined the spatial concordance between each imaging modality and the SEEG‐defined SOZ or resection zone at the patient level. Those results are presented in Methods S3 and Figure S1.

## 4 DISCUSSION

### 4.1 Patient‐level spatial accuracy of MSI, PET, and MRI: Unimodal comparisons

When assessing the spatial concordance with SEEG SOZ and resection zone at the patient level, MSI demonstrated a comparable distribution of full and partial concordance with MRI and PET. At the patient level, there is no major difference in the concordance rates between MSI, PET, and MRI for identifying the SOZ and the resection zone, a result consistent with numerous previous studies.^5^, ^7^, ^8^, ^16^, ^17^, ^18^

The added value of MSI may thus appear limited in the context of a standard non‐invasive presurgical evaluation. This underscores the importance of assessing the complementary contributions of each technique through multimodal analyses at a finer spatial resolution, such as the ROI level.

### 4.2 Multimodal imaging increases the probability of detection of the SOZ and resection zone

The epileptogenic zone is increasingly understood as a network of dynamically interacting regions, a complexity best captured by SEEG recordings.^15^ Although combined multimodal imaging cannot fully resolve the network's fine spatiotemporal dynamics, it can identify multiple key nodes in the epileptogenic network. Few prior studies have evaluated the added value of MSI as part of a multimodal imaging approach. Several reports showed that combining MSI with PET, single‐photon emission tomography (SPECT),^17^ or EEG‐fMRI (functional MRI) improved localization of the epileptogenic zone, particularly in MRI‐negative patients and in specific subgroups such as children^18^, ^19^, ^20^ or operculo‐insular epilepsy.^21^ In temporal lobe epilepsy, PET/MRI combined with MSI also yielded greater accuracy than either modality alone.^22^ However, the absence of ROI‐level analyses in those studies and the lack of MRI and PET post‐processing limit their ability to provide a comprehensive view of the epileptogenic zone.

Our sublobar ROI‐based analysis aimed to assess whether combining imaging modalities improves characterization of the network.

The patient level findings underscore the complementary value of MSI in localizing epileptogenic regions. When positive, MSI identified SOZ regions not captured by conventional imaging in a clinically meaningful proportion of patients: when SEEG was used as the reference standard, MSI identified additional SOZ regions beyond MRI in more than half of patients (55%), and beyond the combined MRI–PET data in one‐third of cases (33%). On a per‐patient basis, this represents unique localizing information that would have been missed had the presurgical workup relied solely on structural or metabolic imaging.

A consistent pattern was observed when the resection zone served as the reference: MSI added new localizing value beyond MRI in 55% of patients, beyond PET in 33%, and beyond the combined MRI–PET approach in 29.6%. Of note, these proportions were preserved in the subgroup of patients achieving Engel class I outcome, strongly suggesting that the additional regions identified by MSI were not incidental findings, but clinically relevant contributions to surgical planning and, ultimately, to seizure freedom.

However, MSI showed discordant findings with both the SEEG‐defined SOZ and the resection zone in 7% to 10% of cases, indicating that, like MRI and PET, it remains susceptible to false‐positive localizations. This highlights that MSI should not independently guide SEEG implantation, but rather be integrated within a multimodal framework alongside the full clinical, electrophysiological, and imaging data.

At the ROI level, diagnostic performance metrics showed that the addition of MSI consistently improved sensitivity for SOZ detection without substantial loss of precision (no drop of PPV). Combining MSI with MRI increased sensitivity, whereas the PET + MSI union outperformed PET alone. The triple combination MRI + PET + MSI achieved the highest sensitivity overall, reaching 82.5% against the surgical standard. GLMM post hoc analyses further demonstrated that combining imaging modalities significantly improved SOZ‐detection rates. The combination of MRI, PET, and MSI outperformed each modality used in isolation, MRI alone (*p* = .003), PET alone (*p* = .008), and MSI alone (p = .008), confirming that multimodal integration provides a statistical gain in localizing yield. Similarly, adding MSI to either MRI or PET individually yielded significant improvements over each modality alone (*p* = .01 and *p* = .05, respectively). Of note, the triple combination MRI + PET + MSI failed to reach statistical superiority over MRI + PET alone, which should be interpreted in light of the limited sample size of this cohort and its inherently constrained statistical power. Moreover, the overlap between modalities yielded high congruence with SEEG‐defined SOZ (up to 99% for MSI combined with MRI and MSI combined with PET), although not significantly different from single modalities after correction.

Combining modalities improved performance for detection of the resection zone, with the combination of MRI, PET, and MSI reaching the highest probability (91%) significantly outperforming MRI alone.

Overall, those results highlight MSI's contribution to refining SOZ and resection zone localization, positioning it as a key complementary tool to MRI and PET for precise delineation of the epileptogenic zone. Adding MSI consistently increased sensitivity for SOZ without introducing additional false positives, an outcome that might have been anticipated given the broader spatial sampling inherent to combined modalities. Our analysis of the F1‐score highlights the inherent trade‐off between sensitivity and spatial precision (PPV) in multimodal imaging. As expected, expanding the detection coverage through union combinations can lead to a reduction in PPV compared to highly specific single modalities. However, the F1‐score remained stable or increased for most MSI‐inclusive unions. This favorable trade‐off indicates that the substantial gains in sensitivity generally outweighed the losses in precision, resulting in a robust overall classification. Although these dynamics are highly consistent with a better non‐invasive characterization of the epileptogenic network, the F1‐score remains a composite metric and should be interpreted as reflecting this clinical balance rather than providing an absolute mathematical proof of isolated added value. Conversely, restricting analyses to overlapping regions across modalities, while ensuring high specificity, did not meaningfully improve the targeting of pathological regions beyond what individual modalities achieved alone. Together, these findings suggest that the main benefit of MSI lies in expanding detection coverage rather than in sharpening spatial precision through multimodal convergence.

It is worth noting that MEG yielded recordable spikes in only 55% of the SEEG cohort, a recognized limitation of the technique. Patients with a negative MSI (i.e., no spike recorded) were considered non‐informative and excluded from the multimodal analysis.

### 4.3 Integrating MSI into the presurgical evaluation of epilepsy: Toward a more comprehensive definition of the epileptogenic zone

Our study highlights the additive value of MSI in a surgically challenging population: patients with non‐ or partially contributive MRI, either normal or showing ambiguous abnormalities such as large, multiple, or lesions of unclear epileptogenic significance. All required SEEG exploration, a marker of poorer prognosis.

Indeed, in these challenging surgical cases, MSI enhances understanding of the epileptogenic network. The combination of MRI and PET represents the most widely used non‐invasive workup strategy for epilepsy worldwide, making it particularly relevant to assess whether MSI provides incremental value to this standard approach. Our findings demonstrate that MSI provides significant added diagnostic value for SOZ detection compared to structural MRI lesions alone, with a somewhat attenuated but still significant improvement when combined with PET, and maintained significance when all three modalities are available. These results are consistent with the inherent characteristics of each imaging technique: MRI reveals structural lesions whose relationship to epilepsy may be uncertain or insufficient when the epileptogenic network extends beyond the visible lesion, whereas PET identifies hypometabolic areas often associated with epileptogenic cortex but whose spatial extent does not directly correspond to the SOZ boundaries. The triple combination therefore maximizes the likelihood of accurately identifying the epileptogenic network. MSI is particularly valuable when MRI and PET are negative or discordant, or when a broad epileptogenic zone is suspected.^9^, ^11^ Because MSI provides added value in identifying the SEEG‐defined SOZ without reducing specificity, it improves the planning of stereo‐EEG electrode placement.^7^, ^23^ Given its non‐invasive nature and efficiency, MSI could be considered in difficult cases, when available, particularly when MRI and PET findings do not allow direct consideration of surgical resection.^24^ Yet, its use remains limited due to high costs, low availability, and lack of reimbursement in many healthcare systems. Looking ahead, the emergence of optically pumped magnetometers (OPMs), more affordable and versatile sensors, could overcome these barriers, paving the way for broader adoption of MSI in routine clinical care.^25^, ^26^, ^27^, ^28^

Several limitations should be acknowledged. First, we must also acknowledge an inherent incorporation bias in our methodology. The reference standard (SEEG SOZ) was not strictly independent of the MSI results, as the non‐invasive multimodal evaluation, including MSI, was discussed during the multidisciplinary conferences to guide SEEG implantation planning. However, although 88% of MSI‐identified regions were explored, these represented only 16% of all implanted regions per patient. This demonstrates that SEEG exploration was substantially broader than the MSI findings alone, being driven by the comprehensive anatomo‐electro‐clinical hypothesis, which minimizes the impact of circular dependency. Second, by restricting our multimodal diagnostic performance analyses exclusively to ‘informative’ (MSI‐positive) cases, we inherently enriched our sample with patients exhibiting detectable interictal activity. This methodological choice may lead to optimistic estimates of MSI's congruence, PPV, and overall added value compared to an unselected cohort. Third, although MSI provides added value for identifying the SOZ as defined by SEEG, overall surgical outcomes remain imperfect, with only ~60% of patients achieving Engel class I, indicating that non‐invasive imaging alone is insufficient to fully delineate the SOZ and resection target. Fourth, the cohort sample size precluded analysis of MSI localizing value according to specific lobar locations, which may exhibit different sensitivity profiles for MSI detection. Finally, more recent post‐processing techniques have been proposed to improve the detection of subtle MRI lesions or hypometabolic foci, and may enhance non‐invasive localization beyond the approaches used in this study.^29^, ^30^

## 5 CONCLUSIONS

This study demonstrates that MSI provides valuable complementary information to MRI and PET in the presurgical evaluation of drug‐resistant focal epilepsy, significantly enhancing the localization of the epileptogenic cortex when used within a multimodal framework. These findings establish MSI as a key imaging modality in the presurgical workup of drug‐resistant epilepsy, particularly valuable when standard imaging techniques fall short of informing surgical planning.

## AUTHOR CONTRIBUTIONS

Julien Jung and François Mauguière designed the current study. Julien Jung and François Mauguière are the scientific coordinators of the EPIMAGE study. Julien Jung, Romain Bouet, and Marc Hermier reviewed and analyzed the data. Julien Jung and Romain Bouet performed the statistical analyses. Julien Jung ensured the coordination of the EPIMAGE study, including the completion and the accuracy of the clinical data required for the study. All authors contributed to the preparation of the report. All authors contributed to the preparation of the report.

## CONFLICT OF INTEREST STATEMENT

The authors declare no conflicts of interest.

## ETHICAL PUBLICATION STATEMENT

We confirm that we have read the Journal's position on issues involved in ethical publication and affirm that this report is consistent with those guidelines.

## Supporting information


Methods S1
Methods S2
Methods S3
Figure S1
Figure S2
Table S1
Table S2
Table S3
Table S4
Table S5

## Data Availability

The data that support the findings of this study are available on request from the corresponding author. The data are not publicly available due to privacy or ethical restrictions.
