# Supplementary material for: Magnetoencephalographic source imaging improves localization of the epileptogenic zone in multimodal imaging evaluation
Source: Epilepsia. 2026 Jun 26;67(9):4717–30. doi: 10.1002/epi.70359 (PMC13592476; doi:10.1002/epi.70359)
Supplement: Supplementary file 1 [file EPI-67-4717-s001.docx]

**Methods S1. Multimodal neuro-imaging data acquisition**

*MRI*

For the visual analysis of the images, all patients underwent brain MRI acquisition on a 3T scanner (Philips Achieva, Philips, Best, The Netherlands), including the following sequences:

(i) 3D anatomical T1-weighted covering the whole brain with 1 mm³ isotropic voxels;

(ii) 6 mm-thick turbo spin-echo T2-weighted acquired in the bi-hippocampal plane;

(iii) 3 mm-thick turbo spin-echo T2-weighted perpendicular to the bi-hippocampal plane;

(iv) 3 mm-thick inversion recovery sequence;

(v) 3D FLAIR volumetric images with isotropic 1 mm³ voxel resolution.

For statistical post-processing only, an additional brain MRI acquisition was performed for all patients, using a 1.5 T Siemens Sonata scanner. A T1-weighted (T1w) anatomical image and a sagittal Fluid-Attenuated Inversion Recovery (FLAIR) sequence were acquired (for T1w: TR = 2400 ms, TE = 3.55 ms, inversion time = 1000 ms, flip angle = 8°; for FLAIR: TR = 6000 ms, TE = 354 ms, inversion time = 2200 ms, flip angle = 180°). Voxel size was 1.2 × 1.2 × 1.2 mm³.

Within 24 hours following electrode implantation, a third MRI (3D T1-weighted sequence) was acquired to precisely localize the recording contacts.

*PET*

PET data were acquired according to recommendations for all included patients, with an activity of 148 ± 2.5 MBq per patient. Data were acquired using a Biograph mCT64 PET/CT (Siemens) and reconstructed with a 3D iterative OSEM algorithm, with CT-based attenuation correction, in a matrix of 200 x 200 x 109 voxels with a voxel size of 2.04 x 2.04 x 2.03 mm³. For statistical post-processing, we used a PET database collected at our center with the same machine and acquisition characteristics in 37 healthy subjects (CERMEP-IDB-MRXFDG database) ^15^.

*MEG*

MEG signals were recorded on a CTF Omega 275 channel whole head system (VSM MedTech Ltd., Canada). For each patient, 45 min of continuous MEG signal were recorded at a sampling frequency of 600 Hz using a third-order spatial gradient noise. Patients were investigated at rest with their eyes closed, without simultaneous EEG acquisition. Three fiducial coils (nasion, left and right pre-auricular points) were placed for each patient to determine the head position within the MEG helmet, and to provide coregistration with the anatomical MR images invasive EEG recordings.

**Methods S2. Analysis of neuroimaging data, SEEG and surgical outcomes**

ROI definition

To enable a meaningful comparison of all neuroimaging maps with a reasonable and consistent anatomical level of detail between modalities and with SEEG, we mapped all significant clusters from MRI, PET, and MSI onto 18 predefined anatomical subregions for each hemisphere.

These subregions include

1 : ventral medial prefrontal (medial orbitofrontal cortex + inferior anterior cingulate gyrus)

2 : dosal medial prefrontal (medial superior frontal gyrus + superior anterior cingulate gyrus)

3 : ventral lateral prefrontal (inferior frontal gyrus)

4 : dorsal lateral prefrontal (lateral superior frontal gyrus + middle frontal gyrus)

5 : medial premotor

6 : lateral premotor

7 : medial centra

8 : lateral central

9 : medial anterior temporal

10 : lateral anterior temporal

11 : medial posterior temporal

12 : lateral posterior temporal

13 : medial parietal

14 : lateral parietal

15 : medial occipital

16 : lateral occipital

17 : operculoinsular

18 : temporo-parieto-occipital junction

MRI

The MRI images from the 3T scanner were reviewed by one expert neuroradiologist (MH) and several neurologists during a multidisciplinary meeting for pre-surgical assessments. The goal was to identify any lesion potentially causing epilepsy in all MRI sequences. The MRI was considered positive if a structural abnormality could explain the electroclinical data.

For additional statistical analysis, T1-weighted and FLAIR images from the 1.5T scanner were processed. The T1 images were analyzed using a voxel-based program (MAP) that detects subtle cortical malformations ^1,2^. MAP highlights abnormal brain regions, focusing on blurred gray-white matter junctions and unusual cortical gyration. The results provide two statistical maps: the junction map (sensitive to blurring at the gray-white matter boundary) and the extension map (sensitive to abnormal cortical extension). These maps identify brain areas that deviate from the normal brain average and may correlate with subtle features of focal cortical dysplasia (FCD). A control database of 70 healthy subjects was used for comparison. In our analysis, we used custom MATLAB scripts within the SPM12 framework to replicate MAP. A z-score map was generated for each feature (junction and extension), and lesions were considered when the z-score exceeded a threshold. We also conducted voxel-wise statistical analysis to confirm these findings, retaining clusters with a p-value < 0.05 (uncorrected p < 0.001) and no minimum cluster size threshold.

For FLAIR analysis, we used a method from Focke et al., which detects hyperintensitis in the brain by comparing each patient’s FLAIR images to the control database ^3^. Similar to the T1 analysis, significant clusters were identified using the same statistical threshold.

To reduce false positives, we only kept clusters that matched localization hypotheses from the electroclinical data and excluded obvious false positives, such as noise, movement artifacts, or non-specific anatomical anomalies.

For each patient, the MRI lesion (referred to as "MRI") was defined as the collection of all ROIs identified by either visual inspection or statistical methods, regardless of the technique used.

*PET*

PET data were independently reviewed by two experienced raters trained in the interpretation of clinical brain [18F]FDG PET coregistered with MRI. Focal hypometabolic regions were identified and assigned to predefined regions of interest (ROIs), as previously described.

In parallel, visual inspection was complemented by a statistical analysis of PET data using a standard SPM pipeline ^4^. Voxel-wise comparisons were performed with SPM12, contrasting each patient's brain PET image against a group of healthy controls. Global signal normalization was applied through an analysis of covariance (ANCOVA), aligning each patient’s global mean uptake to that of the control group. The contrast of interest was patient < controls, reflecting regions of relative hypometabolism. Clusters were considered statistically significant if they comprised voxels exceeding a p-value threshold of <0.001 (uncorrected) and had a cluster-level significance of p < 0.05, with no minimum cluster extent. Significant clusters were mapped to the same set of 18 predefined cortical ROIs.

For each patient, PET abnormalities (referred to as « PET ») were defined as the set of all regions of interest (ROIs) showing hypometabolism, identified either by expert visual assessment or through statistical post-processing methods.

MEG

MEG data were processed using VIES, a pipeline of analysis previously described and validated. VIES aims at localizing the brain spiking volume which generates high frequency activities (> 20 Hz) associated with interictal spikes (see Bouet et al. (2012) for a full description of the method) ^5^.

In VIES, spikes are first visually identified on raw MEG signals. Secondly, the brain sources of spike-related high frequency activity (> 20 Hz) are located using a beamforming technique, DICS (Dynamic Imaging of Coherent Sources) ^6^, comparing the source power for high-frequency during the spike period and a baseline period. The brain sources of spike-related high frequency activity were computed for all 5 mm3 voxels covering the brain volume, providing whole-brain MSI maps. Lastly, the whole-brain MSI maps are thresholded using a method described in Bouet et al. (2012), providing a MSI spiking volume map for each patient.

We retained all brain regions generating interictal spikes based on those maps according to the previously described 18 brain cortical regions (referred to as “MSI”).

SEEG Analysis

For each patient, one SEEG expert (JJ) reviewed all the SEEG-recorded spontaneous seizures visually to define the Seizure Onset Zone (SOZ). It was defined as the cortical area(s) exhibiting clear SEEG changes within the first 10 seconds of the seizure onset. SEEG changes were considered part of the SOZ when they occurred before the clinical onset of the seizure, and when they consisted of a fast-synchronizing discharge (low voltage fast activity, or fast discharge of spikes). The SEEG SOZ (referred to as “SEEG”) was labelled according to the previously described 18 brain cortical regions used for neuroimaging analysis.

Surgical resection

For patients operated upon, the location of the surgical resection was based on detailed surgical reports since post-operative scans were available for a minority of patients. For all patients, surgical reports were sufficiently precise to allocate surgical resection cavity to the 18 cortical ROI (referred to as “Resection Zone”).

The seizure-outcome after surgery was assessed a minimum of one year and follow-up and was based on Engel’s classification ^7^. The outcome was categorized into good (Engel I) or poor (Engel II-III-IV). Pathology reports were collected when available.

**Methods S3. Concordance analysis between unimodal imaging modalities and SEEG-defined SOZ or resection zone at the patient level**

To assess the performance of each imaging modality (MRI, PET, MSI), we systematically evaluated the spatial concordance between the regions identified by imaging and either the seizure-onset zone (SOZ) delineated by SEEG or the resection cavity (for patients who underwent surgery). A concordance metric was computed separately for each modality and comparison (SEEG vs modality; resection zone vs modality), using the following definitions: i) Full concordance: all ROIs in the SEEG SOZ (or surgical zone) were identified by the imaging modality ii) Partial concordance: at least one but not all SEEG SOZ (or surgical) ROIs were identified by the modality iii) No concordance: none of the SEEG SOZ (or surgical) ROIs were present among those identified by the modality. Proportions of full, partial, and no concordance were computed for each modality, both with respect to the SEEG-defined SOZ and the resection zone. Exact binomial confidence intervals (Clopper–Pearson method) were calculated for each proportion using the R package binom.

**Figure S1. Concordance analysis between unimodal imaging modalities and SEEG-defined SOZ or resection zone at the patient level**

(A) Proportions of patients showing full, partial, or no concordance between MRI, PET, or MSI findings and the SOZ as defined by stereo-EEG (SEEG).

(B) Proportions of patients showing concordance between imaging findings and the resected brain region.

Bars represent the proportion of patients for each level of concordance (full, partial, or none), with 95% exact binomial confidence intervals shown as error bars.

Concordance was defined as: full (all SOZ/resected zones included in the imaging-positive areas), partial (some overlap), or none (no overlap).

Each modality was analyzed separately.

For MRI, full concordance was observed in 34.5% of cases (95% CI: 22.2–48.6), partial concordance in 52.7% (95% CI: 38.8–66.3), and no concordance in 12.7% (95% CI: 5.3–24.5). MSI showed equal rates of full and partial concordance (45.0%, 95% CI: 29.3–61.5), while no concordance was found in 10.0% (95% CI: 2.8–23.7). PET yielded full concordance in 42.1% of cases (95% CI: 29.1–55.9), partial in 49.1% (95% CI: 35.6–62.7), and no concordance in 8.8% (95% CI: 2.9–19.3).

We then evaluated how each modality matched the resection zone in operated patients (Figure 5b). Full concordance with MRI was found in 39.5% of cases (95% CI: 24.0–56.6), partial in 55.3% (95% CI: 38.3–71.4), and none in 5.3% (95% CI: 0.6–17.7). MSI results showed full concordance in 55.6% (95% CI: 35.3–74.5), partial in 37.0% (95% CI: 19.4–57.6), and no concordance in 7.4% (95% CI: 0.9–24.3). For PET, full concordance was observed in 51.2% (95% CI: 35.1–67.1), partial in 46.3% (95% CI: 30.7–62.6), and no concordance in only 2.4% of cases (95% CI: 0.06–12.9).

**Figure S2. Multimodal localization of the seizure onset zone (SOZ) in a patient.**

**The figure illustrates the spatial distribution of interictal MEG spikes (green area), the hypometabolic region identified by PET (red area), and the structural lesion detected on MRI (blue area). On the right, SEEG recordings from a selection of representative bipolar derivations at seizure onset are shown, with “SO” indicating the seizure onset.** The SEEG contacts involved at seizure onset (J'1–J'2, A'11–A'12, N'1–N'2, and T'1–T'2) are indicated by yellow boxes.

**The partial overlap and spatial convergence of these modalities emphasize their complementary contribution to accurately delineating the seizure-onset zone (SOZ).**

**Table S1. Patient and imaging details**

The table reports age at surgery, epilepsy duration, sex, findings from magnetoencephalography (MSI), positron emission tomography (PET; visual analysis and SPM), and magnetic resonance imaging (MRI; visual assessment and T1/FLAIR MAP). Imaging results, the seizure onset zone (SOZ) defined by stereoelectroencephalography (SEEG), and the resected area are coded according to an 18-subregion atlas (numbers 1–18, with lateralization R = right, L = left). Non-informative investigations are indicated as UNCONCLUSIVE and patients without surgery as No surgery. Histopathological findings and postoperative outcome are also provided according to Engel’s classification.

Atlas of 18 subregions (codes 1–18):

1. Ventral medial prefrontal (medial orbitofrontal cortex + inferior anterior cingulate gyrus)

2. Medial dorsal prefrontal (medial superior frontal gyrus + superior anterior cingulate gyrus)

3. Lateral ventral prefrontal (inferior frontal gyrus)

4. Lateral dorsal prefrontal (lateral superior frontal gyrus + middle frontal gyrus)

5. Medial premotor

6. Lateral premotor

7. Medial central

8. Lateral central

9. Anterior medial temporal

10. Anterior lateral temporal

11. Posterior medial temporal

12. Posterior lateral temporal

13. Medial parietal

14. Lateral parietal

15. Medial occipital

16. Lateral occipital

17. Operculo-insular

18. Temporo-parieto-occipital junction

Coding rule: Each finding is coded as one or several subregion tokens (e.g., `9L + 10L` = left anterior medial temporal + left anterior lateral temporal)

**Table S2. Probability of detecting each SOZ region of interest (ROI) by imaging modality**

| **Modality** | **Estimated probablity (95 % CI)** | **OR vs MRI (95 % CI)** | **p (FDR-corrected)** |
| --- | --- | --- | --- |
| MRI | 0.64 (0.48–0.77) | Reference | Reference |
| PET | 0.68 (0.53–0.80) | 1.20 (0.66–2.20) | 0.54 |
| MSI | 0.66 (0.50–0.80) | 1.09 (0.56–2.13) | 0.80 |
| MRI U PET | 0.74 (0.60–0.84) | 1.56 (0.87–2.80) | 0.13 |
| MRI U MSI | 0.81 (0.69–0.89) | 2.42 (1.34–4.38) | 0.01 |
| PET U MSI | 0.80 (0.68–0.88) | 2.20 (1.21–4.00) | 0.01 |
| MRI U PET U MSI | 0.85 (0.75–0.92) | 3.22 (1.70–6.10) | <0.001 |

Detection probabilities of each SOZ ROI by each modality were estimated using generalized linear mixed models (GLMMs) with a binomial distribution, including modality as a fixed effect and subject as a random effect (MRI as reference). Values represent estimated detection probabilities (95% CI), odds ratios (OR) versus MRI, and FDR-corrected *p*-values.

| **contrast** | **estimate** | **SE** | **z.ratio** | **p.value** |
| --- | --- | --- | --- | --- |
| MRI - PET | -0.1863 | 0.305 | -0.610 | 0.6318 |
| MRI - MSI | -0.0846 | 0.330 | -0.257 | 0.7975 |
| MRI – MRI U PET | -0.4435 | 0.294 | -1.510 | 0.2294 |
| MRI – MRI U MSI | -0.8821 | 0.305 | -2.897 | 0.0120 |
| MRI – PET U MSI | -0.7880 | 0.302 | -2.608 | 0.0383 |
| MRI – MRI U PET U MSI | -1.1677 | 0.307 | -3.803 | 0.0030 |
| PET - MSI | 0.1017 | 0.327 | 0.311 | 0.7939 |
| PET – MRI U PET | -0.2572 | 0.289 | -0.889 | 0.4619 |
| PET – MRI U MSI | -0.6958 | 0.302 | -2.302 | 0.0560 |
| PET – PET U MSI | -0.6017 | 0.296 | -2.034 | 0.0500 |
| PET – MRI U PET U MSI | -0.9814 | 0.302 | -3.253 | 0.0080 |
| MSI – MRI U PET | -0.3589 | 0.317 | -1.131 | 0.3613 |
| MSI – MRI U MSI | -0.7975 | 0.327 | -2.442 | 0.0438 |
| MSI – PET U MSI | -0.7034 | 0.323 | -2.178 | 0.0687 |
| MSI – MRI U PET U MSI | -1.0831 | 0.329 | -3.292 | 0.0080 |
| MRI U PET – MRI U MSI | -0.4385 | 0.290 | -1.512 | 0.2294 |
| MRI U PET – PET U MSI | -0.3444 | 0.285 | -1.208 | 0.3404 |
| MRI U PET – MRI U PET U MSI | -0.7241 | 0.289 | -2.501 | 0.0433 |
| MRI U MSI – PET U MSI | 0.0941 | 0.296 | 0.318 | 0.7939 |
| MRI U MSI – MRI U PET U MSI | -0.2856 | 0.300 | -0.953 | 0.4468 |
| PET U MSI – MRI U PET U MSI | -0.3797 | 0.295 | -1.286 | 0.3205 |

Post hoc pairwise comparisons (estimated marginal means with false discovery rate correction) of the predicted probabilities for SEEG-defined seizure onset zone detection across imaging modalities (MRI, PET, MSI, and their combinations), derived from a mixed-effects logistic regression model including subject as a random effect.

**Table S3. Concordance between imaging findings and SEEG-defined SOZ by modality**

| **Modality** | **Estimated probablity (95 % CI)** | **OR vs MRI (95 % CI)** | **p (FDR-corrected)** |
| --- | --- | --- | --- |
| MRI | 0.95 (0.84–0.99) | Reference | Reference |
| MSI | 0.88 (0.68–0.96) | 0.37 (0.14–1.01) | 0.06 |
| PET | 0.94 (0.81–0.98) | 0.81 (0.31–2.10) | 0.66 |
| MRI ∩ PET | 0.96 (0.84–0.99) | 1.13 (0.40–3.20) | 0.82 |
| MRI ∩ MSI | 0.99 (0.90–0.99) | 4.78 (0.65–34.9) | 0.14 |
| PET ∩ MSI | 0.99 (0.92–0.99) | 5.65 (0.78–40.9) | 0.09 |
| MRI ∩ PET ∩ MSI | 0.99 (0.90–0.99) | 4.58 (0.58–35.8) | 0.15 |

Concordance between imaging-defined ROIs and the SEEG-defined SOZ was assessed using generalized linear mixed models (GLMMs) with a binomial distribution. The models included imaging modality as a fixed effect and subject as a random effect, with MRI as the reference. The table reports estimated detection probabilities (95% CI), odds ratios (OR) relative to MRI, and FDR-corrected *p*-values.

| **contrast** | **estimate** | **SE** | **z.ratio** | **p.value** |
| --- | --- | --- | --- | --- |
| MRI - MSI | 0.9921 | 0.514 | 1.929 | 0.2018 |
| MRI - PET | 0.2061 | 0.471 | 0.438 | 0.8172 |
| MRI – MRI ∩ PET | -0.1232 | 0.531 | -0.232 | 0.9461 |
| MRI – MRI ∩ MSI | -1.5641 | 1.057 | -1.480 | 0.2431 |
| MRI – PET ∩ MSI | -1.7314 | 1.032 | -1.678 | 0.2227 |
| MRI – MRI ∩ PET ∩ MSI | -1.5222 | 1.067 | -1.427 | 0.2481 |
| MSI - PET | -0.7859 | 0.486 | -1.616 | 0.2227 |
| MSI – MRI ∩PET | -1.1153 | 0.549 | -2.032 | 0.2018 |
| MSI – MRI ∩ MSI | -2.5561 | 1.054 | -2.424 | 0.1280 |
| MSI – PET ∩ MSI | -2.7235 | 1.025 | -2.657 | 0.1280 |
| MSI – MRI ∩ PET ∩ MSI | -2.5143 | 1.066 | -2.360 | 0.1280 |
| PET – MRI ∩PET | -0.3293 | 0.507 | -0.649 | 0.6775 |
| PET – MRI ∩ EG | -1.7702 | 1.049 | -1.687 | 0.2227 |
| PET – PET ∩ MSI | -1.9375 | 1.021 | -1.898 | 0.2018 |
| PET – MRI ∩ PET ∩MSI | -1.7284 | 1.058 | -1.633 | 0.2227 |
| MRI ∩ PET – MRI ∩ MSI | -1.4409 | 1.071 | -1.346 | 0.2677 |
| MRI ∩ PET – PET ∩ MSI | -1.6082 | 1.046 | -1.538 | 0.2369 |
| MRI ∩ PET – MRI ∩ PET ∩ MSI | -1.3990 | 1.080 | -1.296 | 0.2730 |
| MRI ∩ MSI – PET ∩ MSI | -0.1673 | 1.345 | -0.124 | 0.9461 |
| MRI ∩ MSI – MRI ∩ PET ∩ MSI | 0.0418 | 1.367 | 0.031 | 0.9756 |
| PET ∩ MSI – MRI ∩ PET ∩ MSI | 0.2091 | 1.351 | 0.155 | 0.9461 |

Post hoc pairwise comparisons (estimated marginal means with false discovery rate correction) of the predicted probabilities of congruence between imaging-defined regions of interest and the SEEG-defined seizure onset zone across modalities (MRI, PET, MSI, and their intersections), derived from a mixed-effects logistic regression model including subject as a random effect.

**Table S4. Probability of detecting each resection zone by imaging modality**

| **Modality** | **Estimated probablity (95 % CI)** | **OR vs MRI (95 % CI)** | **p (FDR-corrected)** |
| --- | --- | --- | --- |
| MRI | 0.72 (0.55–0.84) | Reference | Reference |
| MSI | 0.73 (0.55–0.86) | 1.09 (0.47–2.55) | 0.85 |
| PET | 0.77 (0.62–0.87) | 1.36 (0.64–2.91) | 0.43 |
| MRI U PET | 0.82 (0.69–0.90) | 1.75 (0.84–3.63) | 0.13 |
| MRI U MSI | 0.87 (0.76–0.93) | 2.60 (1.19–5.66) | 0.016 |
| PET U MSI | 0.86 (0.75–0.93) | 2.50 (1.15–5.44) | 0.020 |
| MRI U PET U MSI | 0.90 (0.82–0.96) | 3.75 (1.68–8.38) | 0.001 |

Detection probabilities of each resection zone ROI by each modality were estimated using generalized linear mixed models (GLMMs) with a binomial distribution, including modality as a fixed effect and subject as a random effect (MRI as reference). Values represent estimated detection probabilities (95% CI), odds ratios (OR) versus MRI, and FDR-corrected *p*-values.

| **contrast** | **estimate** | **SE** | **z.ratio** | **p.value** |
| --- | --- | --- | --- | --- |
| MRI - MSI | -0.0817 | 0.426 | -0.192 | 0.8903 |
| MRI - PET | -0.3044 | 0.385 | -0.791 | 0.5297 |
| MRI - MRI U PET | -0.5614 | 0.375 | -1.499 | 0.2557 |
| MRI - MRI U MSI | -0.9573 | 0.397 | -2.414 | 0.0829 |
| MRI - PET U MSI | -0.9159 | 0.393 | -2.329 | 0.0834 |
| MRI - MRI U PET U MSI | -1.3222 | 0.405 | -3.262 | 0.0232 |
| MSI - PET | -0.2228 | 0.424 | -0.525 | 0.6629 |
| MSI - MRI U PET | -0.4797 | 0.416 | -1.152 | 0.4361 |
| MSI - MRI U MSI | -0.8756 | 0.436 | -2.009 | 0.1387 |
| MSI - PET U MSI | -0.8342 | 0.431 | -1.936 | 0.1387 |
| MSI - MRI U PET U MSI | -1.2405 | 0.444 | -2.797 | 0.0542 |
| PET - MRI U PET | -0.2570 | 0.370 | -0.695 | 0.5681 |
| PET - MRI U MSI | -0.6529 | 0.395 | -1.652 | 0.2301 |
| PET - PET U MSI | -0.6115 | 0.385 | -1.589 | 0.2354 |
| PET - MRI U PET U MSI | -1.0178 | 0.398 | -2.554 | 0.0745 |
| MRI U PET - MRI U MSI | -0.3959 | 0.384 | -1.031 | 0.4664 |
| MRI U PET - PET U MSI | -0.3545 | 0.375 | -0.944 | 0.4831 |
| MRI U PET - MRI U PET U MSI | -0.7608 | 0.387 | -1.966 | 0.1387 |
| MRI U MSI - PET U MSI | 0.0414 | 0.399 | 0.104 | 0.9175 |
| MRI U MSI - MRI U PET U MSI | -0.3649 | 0.409 | -0.891 | 0.4894 |
| PET U MSI - MRI U PET U MSI | -0.4063 | 0.401 | -1.013 | 0.4664 |

Post hoc pairwise comparisons (estimated marginal means with false discovery rate correction) of the predicted probabilities for resection zone detection across imaging modalities (MRI, PET, MSI, and their combinations), derived from a mixed-effects logistic regression model including subject as a random effect.

**Table S5. Concordance between imaging findings and resection zone by modality**

| **Modality** | **Estimated probablity (95 % CI)** | **OR vs MRI (95 % CI)** | **p (FDR-corrected)** |
| --- | --- | --- | --- |
| MRI | 0.97 (0.90 – 0.99) | Reference | Reference |
| MSI | 0.82 (0.63 – 0.92) | 0.16 (0.04 – 0.56) | 0.005 |
| PET | 0.95 (0.87 – 0.98) | 0.68 (0.18 – 2.59) | 0.56 |
| MRI ∩ PET | 0.97 (0.89 – 0.99) | 1.20 (0.25 – 5.68) | 0.82 |
| MRI ∩ MSI | 0.99 (0.85 – 0.99) | 2.80 (0.18 – 44.6) | 0.46 |
| PET ∩ MSI | 1.00 (1.00 – 1.00) | perfect prediction | < 0.001 |
| MRI ∩ PET ∩ MSI | 1.00 (1.00 – 1.00) | perfect prediction | < 0.001 |

Concordance between imaging-defined ROIs and resection zone ROIs was evaluated using generalized linear mixed models (GLMMs) with a binomial distribution. Imaging modality was included as a fixed effect and subject as a random effect, with MRI serving as the reference. The table presents estimated detection probabilities (95% CI), odds ratios (OR) relative to MRI, and FDR-corrected *p*-values.

| **contrast** | **estimate** | **SE** | **z.ratio** | **p.value** |
| --- | --- | --- | --- | --- |
| MRI - MSI | 1.856 | 0.665 | 2.793 | 0.0613 |
| MRI - PET | 0.389 | 0.672 | 0.579 | 1.0000 |
| MRI - MRI ∩ PET | -0.186 | 0.804 | -0.232 | 1.0000 |
| MRI - MRI ∩ MSI | -1.030 | 1.393 | -0.740 | 1.0000 |
| MRI - PET ∩ MSI | -17.817 | 5539.286 | -0.003 | 1.0000 |
| MRI - MRI ∩ PET ∩ MSI | -17.707 | 6312.019 | -0.003 | 1.0000 |
| MSI - PET | -1.467 | 0.576 | -2.548 | 0.0758 |
| MSI - MRI ∩ PET | -2.042 | 0.741 | -2.757 | 0.0613 |
| MSI - MRI ∩ MSI | -2.886 | 1.342 | -2.151 | 0.1651 |
| MSI - PET ∩ MSI | -19.673 | 5539.286 | -0.004 | 1.0000 |
| MSI - MRI ∩ PET ∩ MSI | -19.563 | 6312.019 | -0.003 | 1.0000 |
| PET - MRI ∩ PET | -0.575 | 0.735 | -0.782 | 1.0000 |
| PET - MRI ∩ MSI | -1.419 | 1.378 | -1.030 | 1.0000 |
| PET - PET ∩ MSI | -18.206 | 5539.286 | -0.003 | 1.0000 |
| PET - MRI ∩ PET ∩ MSI | -18.096 | 6312.019 | -0.003 | 1.0000 |
| MRI ∩ PET - MRI ∩ MSI | -0.844 | 1.449 | -0.582 | 1.0000 |
| MRI ∩ PET - PET ∩ MSI | -17.631 | 5539.286 | -0.003 | 1.0000 |
| MRI ∩ PET - MRI ∩ PET ∩ MSI | -17.521 | 6312.019 | -0.003 | 1.0000 |
| MRI ∩ MSI - PET ∩ MSI | -16.787 | 5539.286 | -0.003 | 1.0000 |
| MRI ∩ MSI - MRI ∩ PET ∩ MSI | -16.677 | 6312.019 | -0.003 | 1.0000 |
| PET ∩ MSI - MRI ∩ PET ∩ MSI | 0.110 | 8397.933 | 0.000 | 1.0000 |

Post hoc pairwise comparisons (estimated marginal means with false discovery rate correction) of the predicted probabilities of congruence between imaging-defined regions of interest and the resectio zone across modalities (MRI, PET, MSI, and their intersections), derived from a mixed-effects logistic regression model including subject as a random effect.

REFERENCES

1.	Wang ZI, Jones SE, Jaisani Z, et al. Voxel-based morphometric magnetic resonance imaging (MRI) postprocessing in MRI-negative epilepsies: MAP in Nonlesional Epilepsies. *Ann Neurol*. 2015;77(6):1060-1075. doi:10.1002/ana.24407

2.	Wagner J, Weber B, Urbach H, Elger CE, Huppertz HJ. Morphometric MRI analysis improves detection of focal cortical dysplasia type II. *Brain*. 2011;134(10):2844-2854. doi:10.1093/brain/awr204

3.	Focke NK, Symms MR, Burdett JL, Duncan JS. Voxel-based analysis of whole brain FLAIR at 3T detects focal cortical dysplasia. *Epilepsia*. 2008;49(5):786-793. doi:10.1111/j.1528-1167.2007.01474.x

4.	Bacon EJ, Jin C, He D, et al. Epileptogenic Zone Localization in Refractory Epilepsy by FDG-PET: The Comparison of SPM and SPM-CAT With Different Parameter Settings. *Front Neurol*. 2021;12:724680. doi:10.3389/fneur.2021.724680

5.	Bouet R, Jung J, Delpuech C, et al. Towards source volume estimation of interictal spikes in focal epilepsy using magnetoencephalography. *Neuroimage*. 2012;59(4):3955-3966.

6.	Gross J, Kujala J, Hämäläinen M, Timmermann L, Schnitzler A, Salmelin R. Dynamic imaging of coherent sources: Studying neural interactions in the human brain. *Proc Natl Acad Sci*. 2001;98(2):694-699. doi:10.1073/pnas.98.2.694

7.	Engel Jr J, Van Ness PC, Rasmussen TB, Ojemann LM. *Outcome with Respect to Epileptic Seizures. Engel J Jr: Surgical Treatment of the Epilepsies Ed 2 New York*. Raven Press; 1993.
